# Recent advances in immune checkpoint inhibitors for non-small lung cancer treatment

**DOI:** 10.3389/fonc.2022.1014156

**Published:** 2022-09-27

**Authors:** Reem Altaf, Sarmad Sheraz Jadoon, Syed Aun Muhammad, Umair Ilyas, Yongtao Duan

**Affiliations:** ^1^ Henan Provincial Key Laboratory of Children’s Genetics and Metabolic Diseases, Children’s Hospital Affiliated to Zhengzhou University, Zhengzhou University, Zhengzhou, China; ^2^ Department of Pharmacy, Iqra University Islamabad Campus, Islamabad, Pakistan; ^3^ State Key Laboratory of Esophageal Cancer Prevention and Treatment, School of Pharmaceutical Sciences, Zhengzhou University, Zhengzhou, China; ^4^ Institute of Molecular Biology and Biotechnology, Bahauddin Zakariya University, Multan, Pakistan; ^5^ Department of Pharmaceutics, Riphah Institute of Pharmaceutical Sciences, Riphah International University, Islamabad, Pakistan

**Keywords:** lung cancer, immune checkpoint inhibitors, immunotherapy, non-small lung cancer, chemotherapy

## Abstract

Lung cancer is one of the deadliest types of cancer responsible for thousands of cancer-related deaths. Its treatment has remained a challenge for researchers, but an increase in the knowledge of molecular pathways and biology of lung cancer has dramatically changed its management in recent decades. Immunotherapies and immunomodulation of lung cancer have previously failed for a long time but thanks to continuous research work and enthusiasm, now, this field is emerging as a novel effective therapy. Now, it is hope with potential benefits and promising results in the treatment of lung cancer. This review article focuses on immune checkpoints inhibitors: CTLA-4 inhibitors (ipilimumab and tremelimumab) and PDL-1 inhibitors (durvalumab and atezolizumab) that can be blocked to treat lung carcinoma. It is also focused on critically analyzing different studies and clinical trials to determine the potential benefits, risks, and adverse events associated with immunotherapeutic treatment.

## Introduction

Lung cancer is considered one of the most lethal forms of solid cancer that have destroyed the lives of thousands of people internationally (1). According to WHO, lung cancer is the most common cause of tobacco-related deaths worldwide. Its survival rate is very low with just 5 years of survival in 18% of the patients diagnosed with this disease, which may be attributed to its late diagnosis in advanced stages (2). Generally, it is divided into two main types: small cell lung cancer (SCLC) or non-small cell lung cancer (NSCLC). NSCLC is the major subtype of lung carcinoma that accounts for 85% of the cases, and the available chemotherapies, radiotherapies, and surgical options are less effective against this type of carcinoma (3). In a survey in 2012, it was estimated that 1,800,000 new cases of lung carcinoma were reported, out of which, 1,600,000 died making the death rate of lung cancer 87% in 1 year. It is the second most common cause of cancer deaths in both men and women (4).

Thus, patients having lung cancer should be intervened in its early stage; otherwise, despite all of the treatment strategies, they are not able to survive stage IV of lung cancer (5). Standard chemotherapeutic drugs have been used over the years to treat NSCLC; however, now, advanced therapeutic options are required to overcome the limitations of cytotoxic drugs (6, 7). Even though all of the genetic alterations, especially anaplastic lymphoma kinase (ALK) fusion oncogene mutation and alteration in epidermal growth factor receptor (EGFR) in NSCLC, are well understood, certain limitations still need to be addressed (8–10). Recent research suggests that immune-mediated disruption of carcinogens is more beneficial than the standard chemotherapeutic approach, so immunotherapy is under consideration for lung cancer treatment (11).

## Immunotherapy as a treatment option

The proliferation and malignancy of cancerous cells are linked with both the nature of the solid tumor and their association with the immune system of our body (12, 13). So, immunotherapy is the use of immunotherapeutic drugs that are designed to promote the immune-regulated destruction of cancerous cells. Generally, immunotherapy includes the use of monoclonal antibodies that targets checkpoint inhibitor signals on cancerous cells, immune system activators, vaccines, and cells of the same individual that triggers an immune response (autologous cells). Earlier studies show that immunotherapeutic drugs were not much effective against lung cancer, so the scientist thought that lung cancer is not dependent on our immune system (14). But recent research suggests that lung cancer can destroy our immune system in a variety of ways including the release of inhibitory cytokines, by reduction of chemicals that restrict the stimulation of T cells and by the disruption of major histocompatibility complex antigen expression (15). Nowadays, immunotherapeutic pathways are employed to treat and reduce harmful effects on already treated patients having lung cancer. These pathways include programmed death-1 (PD-1) pathway and the cytotoxic T-lymphocyte-associated antigen 4 (CTLA-4) pathway, T-cell immunoglobulin and mucin domain-containing protein 3 (TIM-3), T-cell immunoreceptor with lg and ITIM domains (TIGIT), and lymphocyte activation gene 3 (LAG-3) (16). **Table 1** enlists the summary of immune checkpoint inhibitors.

**Table 1:** Immune checkpoint inhibitors.

Checkpoint	Binding partner	Receptor expression	Drugs	Trial
**CTLA-4**	B7-1	Effector T cell	Ipilimumab	CheckMate227
	(CD80)	T_regs_	Tremelimumab	CheckMate-568
**PD-1**	(B7-H1)	T cells	Pembrolizumab	CheckMate-057
	PD-L1	TILs, effector	Nivolumab	CheckMate-017
	PD-L2	Regulatory B cells	Atezolizumab	KEYNOTE-010
	(B7-DC)	NK cells	Duravalumab	KEYNOTE-021
			Avelumab	KEYNOTE-024
			PDR001	KEYNOTE-189
			REGN2810	IMpower-131
			Y3300054	IMpower-150
			Tislelizumab	Pembro-RT
			Mga012	CT02008227
			MEDI4736	NCT02125461
			SHR-1210	NCT02395172
			AB122	
**LAG-3**	MHC0-II	Effector T cells	LAG	NCT03250832
	Galectin-3	T_regs_, B cells	TSR-033	NCT02460224
	LSECtin	NK-cells, DCs	BMS-986016	NCT01968109
	A-synuclein FGL1		REGN3767	NCT02966548
**TIM-3**	Galectin-9	Effector T cells	LY3321367	NCT03099109
	Ceacam-1	B cells, T_regs_	BGB-A425	NCT03744468
	HMGB-1	DCs. NK cells	MBG453	NCT02608268
	PtdSer	Monocytes	TSR-022	NCT02817633
**TIGIT**	CD155	T cell, NK cells	Domvanalimab	AB154
	CD112	NK cells	Tiragolumab	MTIG7192A

CTLA-4, cytotoxic T-lymphocyte antigen-4; PD, programmed death 1; PD-L1, programmed cell death ligand-1; LAG, lymphocyte-activation gene 3; TIM-3, T-cell immunoglobulin and mucin domain 3; TIGIT, T-cell immunoreceptor with immunoglobulin and ITIM domain.

## CTLA-4 receptor

**Figure 1:**
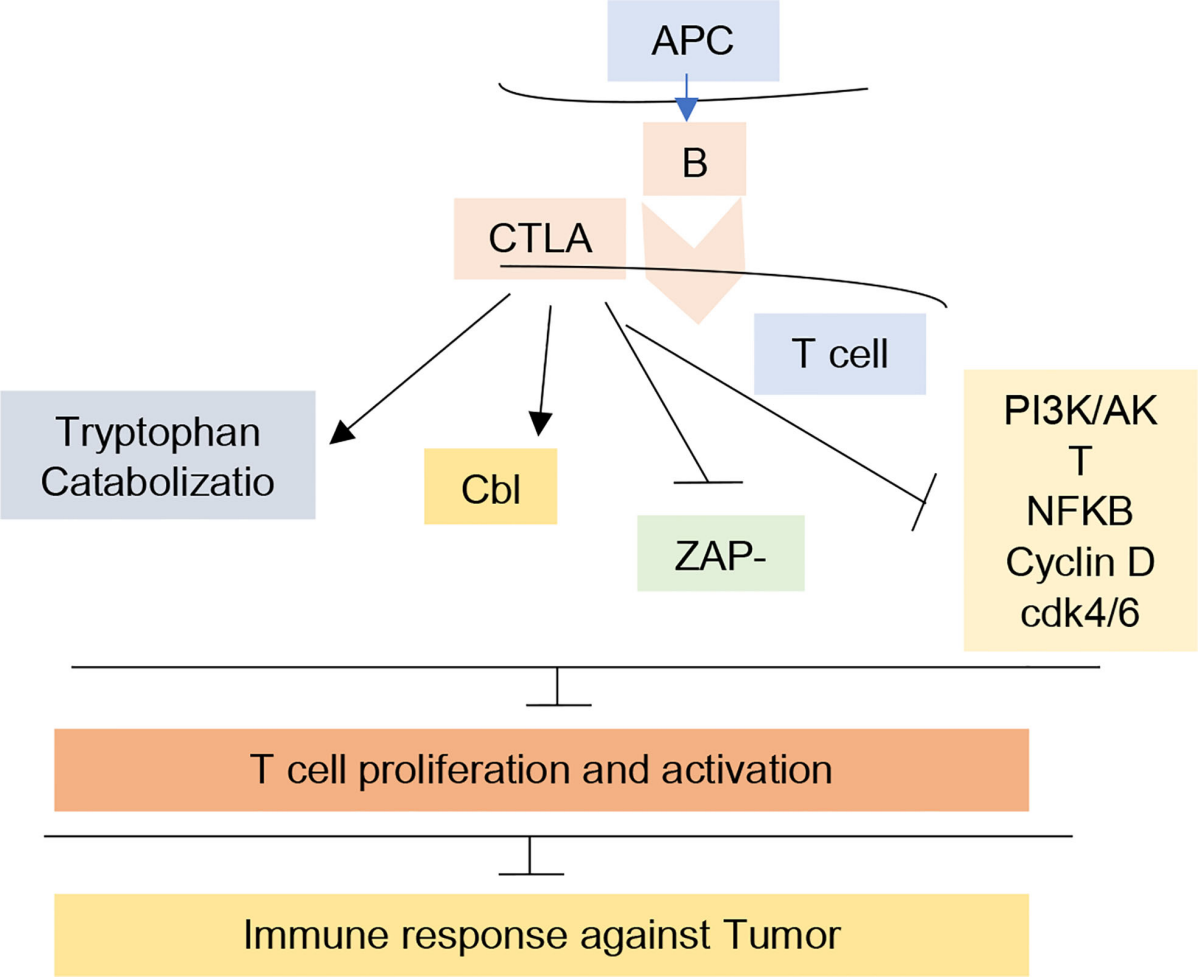
CTLA-4 mechanism for T-cell regulation. The figure illustrates the relation of CTLA-4 with Tryptophan catabolization, Cbl, ZAP-70, and PI3K pathways leading to inhibition of T-cell proliferation and activation and inhibited immune response against tumor. In cancer immunotherapy, CTLA-R receptor was the first receptor to be targeted clinically. Blockade of this receptor by antibodies prevent its interaction in immune system inhibition.

**Figure 2:**
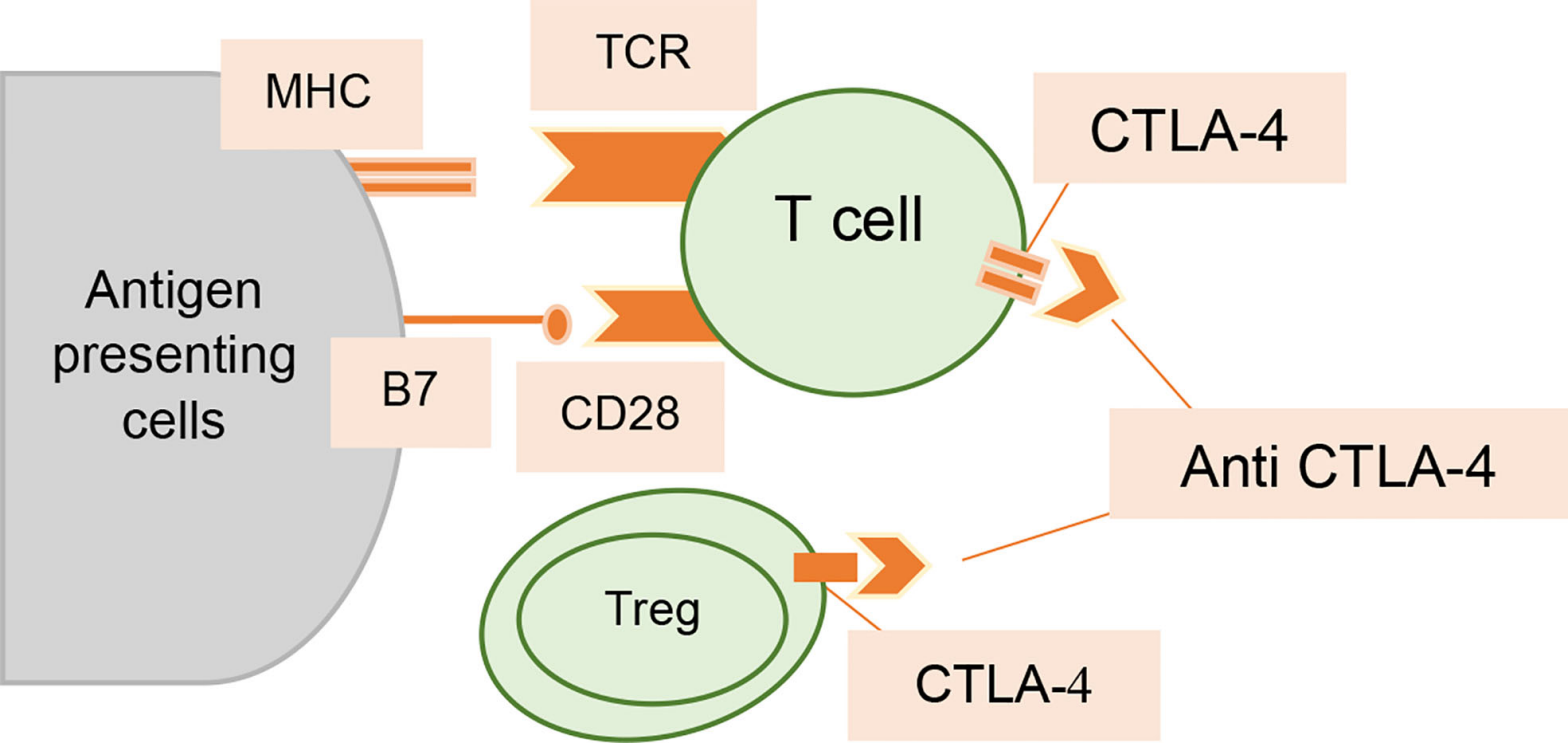
Interaction of anti-CTLA-4 antibodies with T cells. Increased T-cell activation and proliferation are made possible by CTLA-4 inhibition; it also lessens the immunosuppressive effects of T_regs_.

CTLA-4 is a membrane receptor expressed on T cells that regulates the activation of T cells. It is a CD28 homolog and binds with B7 but with more affinity than CD28. However, its binding with B7 does not produce stimulatory signals, rather it produces inhibitory signals (17). It has a negative impact on T-cell activation by following mechanisms (18, 19) (1). Following T-cell receptor (TCR) activation, CTLA-4 is upregulated and more avidly binds B7 than T lymphocyte receptor CD28, which inhibits T-cell proliferation and lowers cytokine release (20) (2). By generating inhibitory signals to diminish the immune response against the tumor in the early stages of carcinogenesis, CTLA-4 could reduce T-cell activation (3). CTLA-4 activates B7 to induce indoleamine-2, 3-dioxygenase (IDO), which catabolizes the amino acid tryptophan and consequently inhibits the proliferation of T cells (21, 22) (4). It has been suggested that CTLA-4 negatively regulates T-cell activation by suppressing the production of the zeta-associated protein (ZAP 70) or promoting the expression of Casitas-B-lineage lymphoma (Cbl) protein (23) (5). According to recent research, CTLA-4 also inhibits The PI3K/Akt pathways, cyclin D3, cyclin-dependent kinases (cdk4/cdk6), and nuclear transcription factors (NF-B) (24) (6) by increasing the movement of T cells, thus reducing the chance of T cells’ interaction with antigen-presenting cells (25) (**Figures 1**, **2**).

## CTLA-4 inhibitors

### Ipilimumab

Ipilimumab is the most recognized inhibitor of CTLA-4. Ipilimumab is the first ever approved checkpoint inhibitor for the treatment of cancer. It is a fully humanized immunoglobulin monoclonal antibody that blocks the interaction of CTLA-4 with its ligand, thus blocking CTLA-4’s negative impact on T-cell regulation. Through this mechanism, ipilimumab allows the attack of immune system on tumor cells (26). Ipilimumab was first patented by University of California (27). However, ipilimumab monotherapy is not successful in treating non-small cell lung carcinoma. Thus, it is combined with other anticancer drugs I-e paclitaxel plus carboplatin with ipilimumab or ipilimumab with other immune checkpoint inhibitors (28). Ipilimumab shows promising results in prolonging the overall survival rates in the patients with advanced melanoma. Phases II and III clinical trials showed satisfactory survival rate of up to 3 years in almost 26% of the population when treated with ipilimumab (29, 30). However, this immune blockade is also associated with some adverse events including rashes, gastrointestinal tract abnormalities (inflammation of colon and diarrhea), pituitary gland inflammation (2% to 4%) (31), endocrine gland disorders (32), and effects on skin, kidney, liver, nervous system, and pancreas. Rash is considered as the characteristic adverse event of ipilimumab that does not occur while treating with any other anticancer agent (33).

Recent research reveals that anti–CTLA-4 antibodies may cause a severe and extensive form of inflammatory bowel illness, which recommends that patients receiving anti–CTLA-4 therapy should avoid using nonsteroidal anti-inflammatory medicines (NSAIDs).

These adverse effects may be associated with the expression of CTLA-4 receptors on pituitary gland leading to binding of ipilimumab with the receptor and induction of inflammation and adverse events and activation of the non-tumor specific T cells. Most of these effects are rare and manageable I-e effect of ipilimumab on liver, and endocrine system is less common (34). The risk of ipilimumab associated adverse events is dose dependent. A study conducted by Bertrand et al. showed that at 3mg/kg dose, 61% of the patients were having adverse events, whereas by increasing the dose to 10mg/kg, the rate of adverse events was increased up to 79% (35).

### Tremelimumab

Tremelimumab is a fully humanized immunoglobulin monoclonal antibody (36). Tremelimumab prevents the interaction of CTLA-4 with its ligand, thus inhibiting its negative regulation that in turn reduces the regulatory T cell (T_reg_)–mediated suppressive response and activates the production and differentiation of T cells (37).

## PD-1 receptor

PD-1 is a Type-1 integral polytopic protein that is regulated by the PDCD1 gene (38). It belongs to the family of CD28/CTLA-4 immunoglobulin antibodies and limits the expression of immune-mediated T cells. Based on the structure, it is divided into three main regions: an intracellular domain, an extracellular region containing IgV, and an aquaphobic cytoplasmic domain (39).

PD-1, a transmembrane protein has two ligands: PD-L1 and PD-L2. Both of these ligands work together to inhibit the immune system in response to external stimuli. PD-L1 is present on B cells, macrophages, resting T cells, vascular endothelial cells, and on islets of Langerhans in the pancreas, whereas PD-L2 is expressed only on dendritic cells (DCs) and on macrophages. As a result, it is less dominant in tumor cells as compared with PD-L1 ligand (40, 41).

## Management, control, and expression of programmed death-1 (PD-1/PD-L1) pathway

In NSCLC, the expression of PD-L1 is regulated by a variety of inflammatory chemicals such as TNF-α, VEGF, cytokines IL-4, and IL-10 along with the most powerful chemical: IFN-γ (42, 43). The overexpression of PD-L1 in the surroundings of cancerous cells stimulates the inhibition of immune response and produces an “acquired immunity resistance” effect. In normal immune response, activated T cells produce interferon-gamma to kill the antigen, but an enhanced expression of PD-L1 in the micro-environment of cancerous cells promotes protection and proliferation of cancer cells (44).

In normal cells, the induction of PD-L1 is restricted by a gene named PTEN (phosphatase and tension homolog deleted on chromosome 10) gene. In lung cancer, the PTEN gene gets altered repeatedly. This mutated PTEN gene stimulates the S6K1 gene (45), enhances the PD-L1 mRNA translation, and causes overexpression of PD-L1 in the plasma membrane (46).

Generally, PD-1 restricts our immune system and induces self-resistance in response to infection (47). In cancerous cells, enhanced expression of PD-L1 and PD-L2 promotes their binding to activated T cells and restricts T-cell stimulation. This will facilitate the escape of the tumor from the T-cell invasion and promotes its proliferation and multiplication (48).

## Immunotherapeutic drugs against programmed death-1 (PD-1) pathway

### Immunoglobulins that target PD-1 transmembrane protein

#### Pembrolizumab

It is a type of immunoglobulin that is formed in the laboratory by the fusion of fragments of antibodies obtained from humans and rats and is monoclonal. It shows a high affinity toward PD-1 transmembrane protein and selectively binds with it. PD-1 protein has an alteration at C228P that is aimed at reducing Antibody-Dependent Cellular Cytotoxicity (ADCC) regulated by the Fc receptor on the surface of the antibody. The safety profile of Pembrolizumab and its activity against hard tumors is still under clinical research trials (49).

#### Nivolumab

It is an IgG4-humanized monoclonal immunoglobulin that is deprived of antibody-dependent cellular cytotoxicity (ADCC) and is sold under the brand Opvido. It binds with PD-1 transmembrane and restricts the activity of PD-1 on tumor-infiltrating lymphocytes (*TIL*) inside the cancerous cells of the lungs. This will prevent the escape of cancerous cells from our immune system through the production of T cells and the release of cytokines (50).

On 4 March 2015, U.S. Food and Drug Administration (FDA) has approved the safety and efficacy of nivolumab against NSCLC (49).

#### Atezolizumab

Atezolizumab is a fully humanized immunoglobulin monoclonal antibody that has a modified Fc domain that prevents the binding of Fc-receptor (51). Atezolizumab treatment has also demonstrated superior results in various cancer types with high levels of PD-L2 expression (52). In a phase 1b trial conducted to determine its safety, atezolizumab and chemotherapy were combined as the first line of treatment for NSCLC patients. Patients got atezolizumab in addition to 4–6 doses of chemotherapy that was platinum based, then atezolizumab as a maintenance treatment was given. Grades 3–4 toxicity was found in up to 13% of patients, with most of these cases being hematological and connected to chemotherapy. There was one recorded death from candidemia following an extended neutropenia (53). In a phase I clinical study with atezolizumab, the most common adverse effects were found to be dehydration, restlessness, shortness of breath, and pericardial effusion. No death was reported in this study. Regardless of PD-L1 expression, atezolizumab has recently been tested in combination with chemotherapy and bevacizumab in patients with metastatic non-squamous NSCLC that has not previously been treated.

#### Durvalumab

Durvalumab currently being evaluated for multiple malignancy treatment is an IgG1 monoclonal antibody that shows high affinity toward PD-L1 receptor. Durvalumab has a distinctive immunomodulatory activity and is potentially used as immune checkpoint inhibitor in cancer immunotherapy. In 2017, in United States, durvalumab was approved for advanced urothelial cnacer but was withdrawn in 2021 due to failure in improvement in survival. The indications were then expanded to include refractory NSCLC and SCLC. Some common side effects are associated with durvalumab including fatigue, headache, pain, diarrhea, weight loss, and so forth (54).

### Immunoglobulins that block the PD-L1 pathway

An alternative treatment methodology that involves binding the therapeutic antibody to the PD-L1 receptor instead of PD-1 transmembrane protein is now introduced to restrict PD-1 activity. This targeted therapeutic method is more beneficial as compared with the inhibition of PD-1 protein, as it will reduce the immune response only in the surroundings of the cancerous cells. Hence, less damage to the normal cells occurs. Following antibodies are used, which selectively bind with the PD-L1 receptor.

#### MEDI4736

It is IgG1 immunoglobulin that is humanized in nature and binds specifically with the PD-L1 receptor. The Fc region of MEDI4736 is made in such a way that it will not produce

ADCC (antibody-dependent cell cytotoxicity) (55). Recent research suggests that its frequent use will help to decrease the size of the tumor even in the initial phase of treatment (56).

#### BMS-936559

It is highly specific IgG4 monoclonal immunoglobulin that is fully obtained from a human source and selectively binds with the PD-L1 receptor to restrict the attachment of PD-1 and PD-L1 to T cells of our immune system (57).

#### LAG-3

LAG-3 (lymphocyte-activation gene 3) having the lg-like domains 1–4 was discovered in 1990 and is also known as CD223. One of the domains of LAG-3, the domain 1 consists of additional ~30 sequence of amino acids, which is termed as extra loop. The cells expressing LAG-3 include the CD4^+^ and CD8^+^ T cells. The activated B cells, T_regs_, plasmocytoid DCs, and NK T cells have also shown expressions of cell surface LAG-3. The potential ligands discovered for LAG-3 are the stable peptide-major histocompatibility complex class II (MHC-II), galectin-3, liver sinusoidal endothelial cell lectin (LSECtin), and fibrinogen-like protein 1. The tumor escape mechanism for lag-3 is similar to that of PD-1 acting as a crucial tumor therapeutic target after PD-1. Work is in progress to verify the effectiveness of LAG-3 immune checkpoint inhibitors either alone or in combination in clinical trials (58).

#### TIM-3

**Figure 3:**
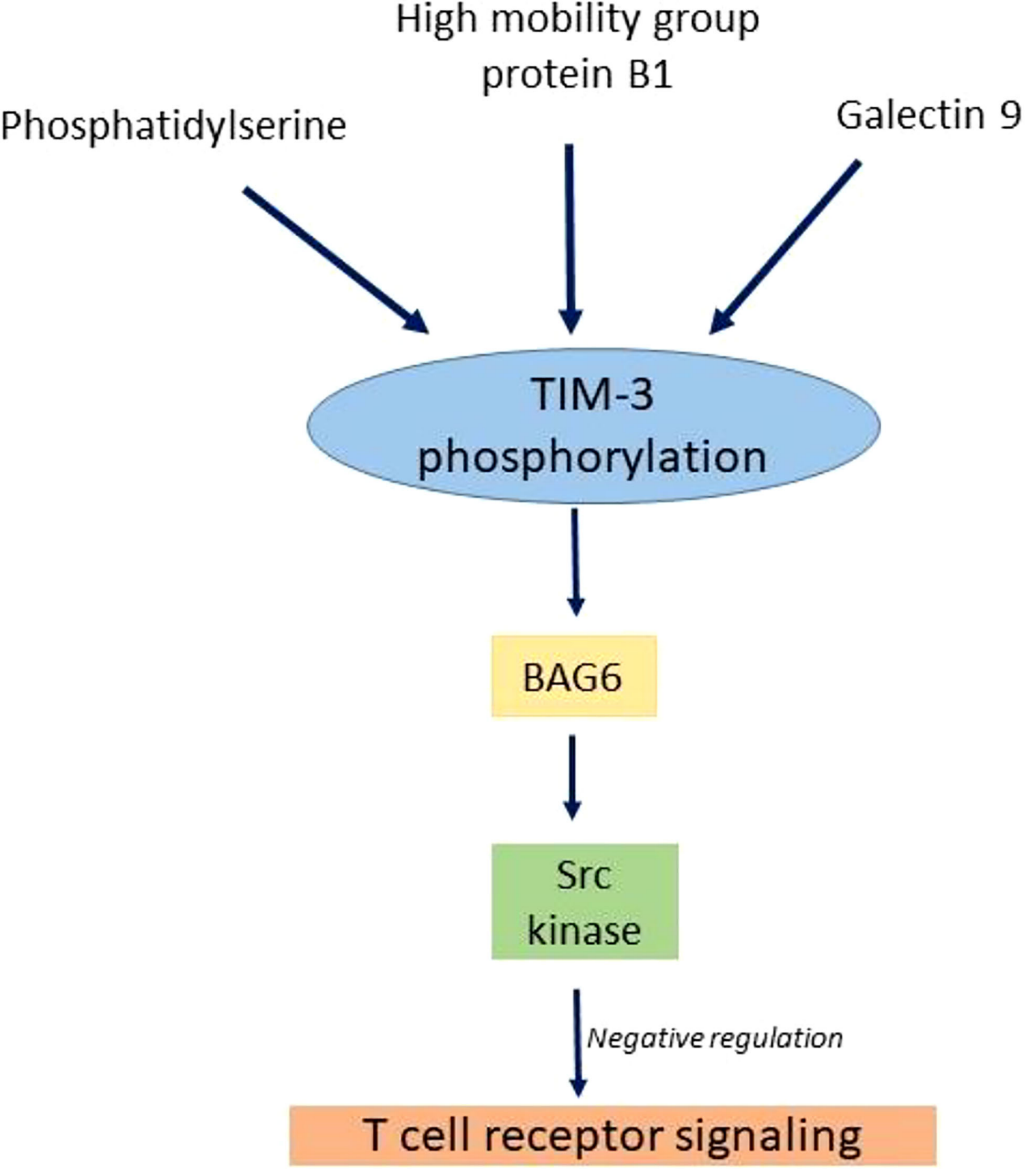
Mechanism of inhibition of TIM3 pathway by ligands.

The TIM family of immunoregulatory proteins TIM-3 (T-cell immunoglobulin and mucin domain 3), a type I transmembrane protein, was first discovered in 2002 and have shown expression on T cells, NK cell, B cells, monocytes, and DCs (58). The ligands reported for TIM-3 and their mechanism have been summarized in **Figure 3**.

#### TGIT

**Figure 4:**
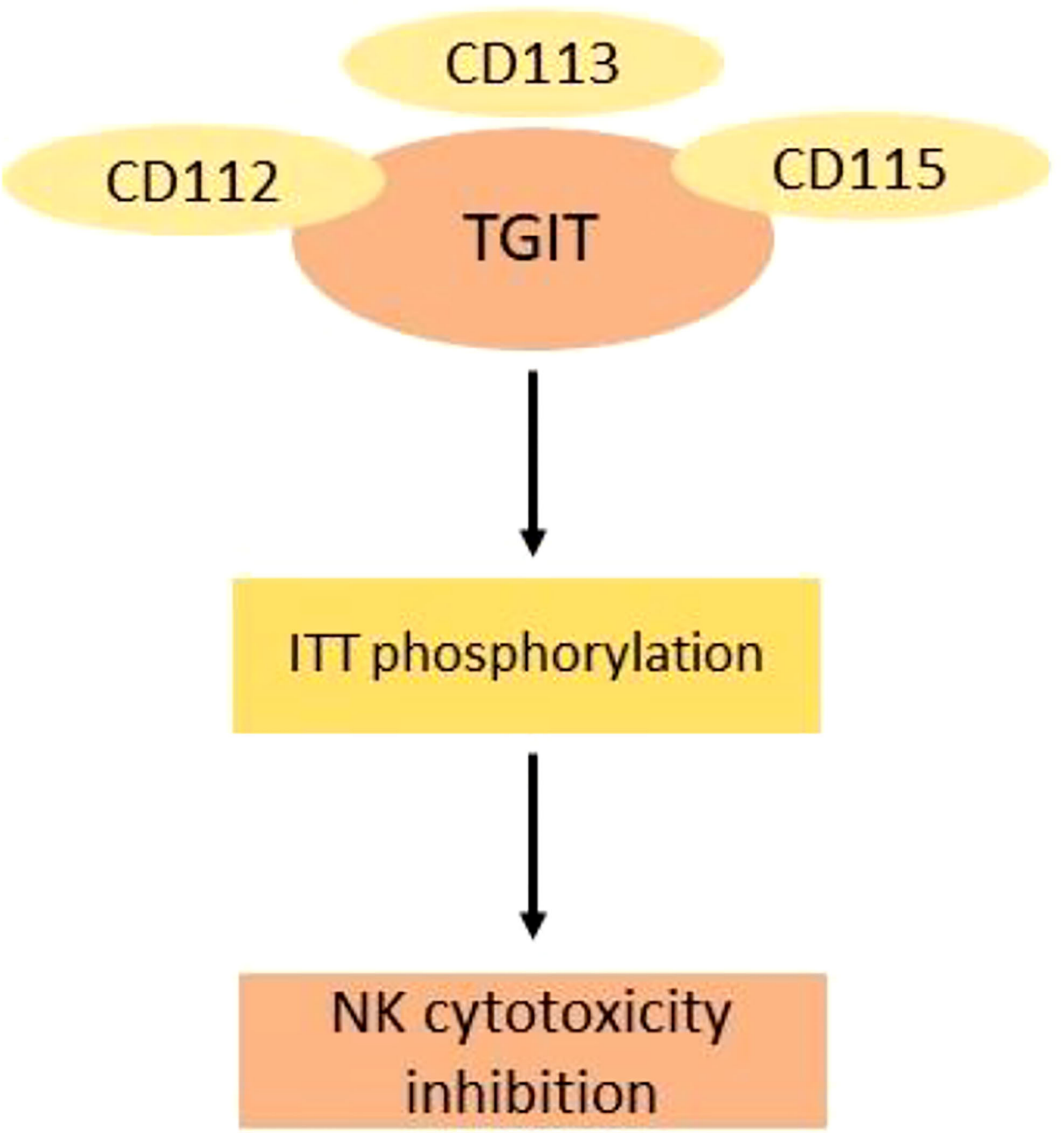
Mechanism of inhibition of cytotoxic NK cells by TGIT ligands. The binding of TGIT with its ligand in NK cells is inhibited by phosphorylation of ITT. CD115 has the highest affinity for TGIT.

TIGIT (T-cell immunoreceptor with immunoglobulin and ITIM domain) belongs to a poliovirus receptor-like protein family and is also called as WUCAM, Vstm3, and VSIG9. These proteins have an important role in restricting immune-related processes. TGIT was first discovered in 2009 and have an extracellular immunoglobulin variable domain. It belongs to the groups of checkpoints that are involved in the inhibition of NK and T-cell activation. The domains of TGIT are the transmembrane domain type 1, a cytoplasmic tail having two inhibitory motifs, one inhibitory motif of immunoreceptor tyrosine based, and one immunoglobulin tyrosine tail (ITT)–like motif. The expression of TGIT is found in activated CD4^+^ T and CD8^+^ T cells, follicular helper cells, NK cells, and T_regs_ (58) (**Figure 4**).

## Combination therapy for NSCLC

A novel approach of combination therapy has been employed nowadays to increase the clinical effectiveness of immunotherapeutic drugs and reducing their toxicity (59, 60). This involves the combination of PD-1/PD-L1 inhibitors with other chemotherapeutic drugs such as anti-angiogenic inhibitors, platinum-based cytotoxic drugs, immunomodulatory cytokines (IFN-y), and inhibitors of immune system checkpoints (61, 62). Research suggests that the combination of PD-1/PD-L1 inhibitors with immunomodulatory drugs will provide the most promising therapeutic results in NSCLC patients (63).

Therapies targeting the CTLA-4 and PD-1 receptors act on different receptors; thus, they have the potential to be used simultaneously for the synergistic action to treat advanced cancer. A study conducted by Das et al. showed that proliferative signal induced by CTLA-4 inhibition was found in transitional memory T cells, whereas PD-1 inhibition was connected to changes in genes thought to be involved in cytolysis and natural killer cell function. Thus, the dual blockade resulted in nonoverlapping changes in gene expression (64). Both anti–CTLA-4 and anti–PD-1 compounds have different impact on the level of cytokines. Preclinical studies have also showed the enhanced anticancer effect of dual therapy of anti PD-1 and anti CTLA-4 in comparison with single agent treatment (65).

Anti–PD-1 along with anti–CTLA-4 inhibitors offer superior toxicity profiles against lung cancer cells, longer overall survival rates, and higher objective response rates when compared with chemotherapy (66). Thus, it is the new standard of care for patients with newly diagnosed advanced NSCLC, provided they are in generally good health and do not have any contraindications to immunotherapeutic treatment (67).

IMpower 130 was evaluated in a study for the therapeutic efficacy of platinum-based chemotherapy in combination with atezolizumab for advanced NSCLC showing improved OS (overall survival) and PFS (progression free survival) in the combination group as compared with the treatment with chemotherapeutic agent alone (68). From this study, the U.S. FDA also approved the combination therapy of chemimmunotherapy with antiangiogenic agent for treating the nonsquamous NSCLC. The combination therapy including carboplatin-paclitexel-atezolizumab and bevacizumab also showed better OS and PFS (69). In another study, Keynote 189, promising therapeutic effects were observed for the chemimmunotherpay as compared with chemotherapy alone in NSCLC and were also the first clinical trial for chemimmunotherapy. The combination pembrolizumabd and platinum-based chemotherapy was used showing improved OS and PFS of NSCLC patients (70).

## Discussion and conclusion

After decades of hard work and several researches, now, immunotherapy has become a reality, an effective treatment for treating lung carcinoma, and to eradicate cancer cells. Blockade of immune checkpoints has transformed lung cancer treatment. They have proven themselves more efficacious than the standard of care chemotherapeutic treatment. This treatment treats not only NSCLC but also SCLC in its extensive stage (either in the form of monotherapy or in combination). However, like other anti-cancer drugs, they have adverse effects (71). The adverse effects associated with immunotherapy are as a result of inflammation in response to immune hyperactivation. These adverse events are collectively termed as the immune-related adverse events (irAEs). Four probable mechanisms have been described for irAEs, but the exact pathophysiology involved in these events is unknown. The enhancement of preexisting antibody levels due to modulation of humoral immunity, the cross reactivity of T cells because of similar antigen, increase in the number of inflammatory cytokines, and the complement-mediated inflammation enhancement are the possible mechanisms leading toward the adverse events due to immunotherapy (72). For example, CTLA-4 inhibition can cause inflammation of colon, whereas PD-1 inhibition leads to pneumonitis that can be fatal. Because patients with lung cancer usually have low lung reserve due to current or past smoking history, pneumonitis is a particular concern in this population. Pneumonitis can thereby further worsen an already inadequate lung reserve and, in extreme situations, even result in death. Despite this worry, pneumonitis is an uncommon occurrence. In case of severe adverse effects, it is necessary to stop the immunotherapy or treatment with corticosteroid is essential (73). The meta-analysis on nivolumab by Zhao et al. proved a low risk of adverse effects when compared with the chemotherapy; however, some of them can be fatal and cannot be ignored (74).

Keeping in view their benefits in treating lung carcinoma, their future either in combination or as monotherapy seems to be bright in lung carcinoma. However, their true potential can be determined by additional studies with anti–PD-1 receptors. The future research can be focused on determining the answers to questions such as determination of the mechanism of immunotherapy resistance. Although the road to effective immunotherapy for lung cancer has not been easy, both animal and human researches have taught us important insights. Thanks to a greater understanding of lung cancer immunoescape and immunosubversion, together with cancer immunosurveillance, immunoediting, and methods to reawaken cancer immunity, we may now embark on a future full of possibilities in using immunotherapy as effective lung cancer treatment.

## Author contributions

SM and UI: Study design. RA and SS: Conceptualization, Study design. YD: Project supervision, administration. All authors contributed to the article and approved the submitted version.

## Conflict of interest

The authors declare that the research was conducted in the absence of any commercial or financial relationships that could be construed as a potential conflict of interest.

## Publisher’s note

All claims expressed in this article are solely those of the authors and do not necessarily represent those of their affiliated organizations, or those of the publisher, the editors and the reviewers. Any product that may be evaluated in this article, or claim that may be made by its manufacturer, is not guaranteed or endorsed by the publisher.
